# In Vivo Hematopoietic Stem Cell Genome Editing: Perspectives and Limitations

**DOI:** 10.3390/genes13122222

**Published:** 2022-11-27

**Authors:** Nikoletta Psatha, Kiriaki Paschoudi, Anastasia Papadopoulou, Evangelia Yannaki

**Affiliations:** 1 Department of Genetics, Development and Molecular Biology, School of Biology, Aristotle University of Thessaloniki, 54124 Thessaloniki, Greece; 2 Gene and Cell Therapy Center, Hematology Clinic, George Papanikolaou Hospital, Exokhi, 57010 Thessaloniki, Greece; 3 Department of Hematology, School of Medicine, University of Washington, Seattle, WA 98195, USA

**Keywords:** in vivo genome editing, hematopoietic stem cells, CRISPR/Cas9, AAV, adenoviral vectors, epigenome editing

## Abstract

The tremendous evolution of genome-editing tools in the last two decades has provided innovative and effective approaches for gene therapy of congenital and acquired diseases. Zinc-finger nucleases (ZFNs), transcription activator- like effector nucleases (TALENs) and CRISPR-Cas9 have been already applied by ex vivo hematopoietic stem cell (HSC) gene therapy in genetic diseases (i.e., Hemoglobinopathies, Fanconi anemia and hereditary Immunodeficiencies) as well as infectious diseases (i.e., HIV), and the recent development of CRISPR-Cas9-based systems using base and prime editors as well as epigenome editors has provided safer tools for gene therapy. The ex vivo approach for gene addition or editing of HSCs, however, is complex, invasive, technically challenging, costly and not free of toxicity. In vivo gene addition or editing promise to transform gene therapy from a highly sophisticated strategy to a “user-friendly’ approach to eventually become a broadly available, highly accessible and potentially affordable treatment modality. In the present review article, based on the lessons gained by more than 3 decades of ex vivo HSC gene therapy, we discuss the concept, the tools, the progress made and the challenges to clinical translation of in vivo HSC gene editing.

## 1. Introduction

For the last two decades, the development of an expanding set of genome editing tools is creating novel prospects in the field of gene therapy. The most extensively studied genome editing technologies include the Zinc-Finger Nucleases (ZFNs), the transcription activator-like effector nucleases (TALENs) and the Clustered Regularly Interspaced Short Palindromic Repeats (CRISPR)-Cas9 nuclease (CRISPR-Cas9). A common feature of all these genome editing approaches is the precise modification of a specific DNA locus via artificial, programmable nucleases which produce double-strand breaks (DSBs) to a predetermined target genome site. The introduction of DSBs leads to the activation of endogenous DNA repair mechanisms: non-homologous end joining (NHEJ), homologous directed repair (HDR) or microhomology-mediated end joining (MMEJ). The most usually activated repair mechanism in human cells is NHEJ [1], which acts in an error-prone manner introducing small deletions and/or insertions (Indels) to the targeted genomic region, eventually disrupting the open reading frame. The HDR mechanism is more precise and requires cell cycling and a DNA donor-sequence as the repair template [2]. These site-specific modifications could lead to permanent genetic changes including the repair of a non-functional gene, the replacement of a missing/dysfunctional gene or an interference with gene expression.

Gene therapy and genome editing of hematopoietic cells have been explored mostly as an ex vivo approach. Ex vivo HSC genome editing is a personalized therapy requiring the production of a unique therapeutic product for each patient, generated from the patient’s own HSCs. The process involves patient mobilization and HSC collection, cell manipulation through electroporation or viral transduction, patient myeloablation and transplantation of the edited autologous graft. The multiple steps of this personalized therapeutic approach requiring specific infrastructure and transplantation expertise add to the complexity of the approach, thus increasing the possibility of an introduction of human error and limiting commercial access to the product due to exorbitant overall costs. Notwithstanding ex vivo gene therapy via gene addition or genome editing has, in recent years, led to unprecedented successes, it remains not broadly available and accessible to patients in need.

Towards overcoming current hurdles of ex vivo gene therapy/editing, in vivo approaches for HSC gene therapy have been proposed that would significantly simplify and expedite the delivery process, reduce cost and offer universal accessibility to patients. In vivo genome editing in non-hematopoietic tissues has been studied and well-argued in preclinical and clinical studies [3,4] whereas in vivo HSC genome editing is less well studied.

In this article, we briefly review the successes of ex vivo HSC genome editing and discuss the current status and the prospect of in vivo HSC gene editing as a therapeutic platform.

## 2. Genome Editing Modules and Ex Vivo HSC Gene Therapy

The development of genome editing techniques created new possibilities for gene correction and genomic modification of hematopoietic stem cells for several genetic and non-genetic disorders including β-hemoglobinopathies, primary immunodeficiencies (PIDs), congenital cytopenias and HIV.

## 3. ZFNs

Zinc-fingers nucleases (ZFNs) were amongst the first genome editing tools applied in HSCs. ZFNs are artificial recombinant nucleases composed of a Zn finger DNA-binding protein domain, fused with the endonuclease domain of the Fok1 restriction enzyme. The specificity of DNA targeting relies on a complex nucleotide-amino acid interaction code. The protein domain consists of three or more tandem zinc-finger individuals, each of which recognize three base pairs. Given that Fok1 dimerizes for activation, the binding of ZFNs pair to a specific DNA locus, resulting in the generation of a double-strand break (DSB) and the subsequent activation of endogenous DNA repair mechanisms [5,6].

Zinc-finger nucleases have been employed to target a variety of genomic loci as a curative means for both β-thalassemia and sickle cell disease. In combination with a single-stranded oligodeoxynucleotide (ssODN) as a donor template, ZFNs have been applied for an HDR-mediated correction of the SCD mutation in HSCs derived from SCD patients [7]. As co-inheritance of hereditary persistence of fetal hemoglobin (HPFH) with both β thalassemia and sickle cell disease (SCD) has been shown to ameliorate the severity of symptoms, ZFNs have been employed to reactivate the expression of the developmentally silenced fetal hemoglobin (HbF). Specifically, a ZFN-mediated inactivation of BCL11A, a major “silencer” of HbF expression, efficiently induced γ-globin expression in thalassemic HSCs [8,9,10]. The important identification of the erythroid enhancer of *BCL11A*, composed of three functional elements in +55 kb, +58 kb and +62 kb, made possible the functional disruption of BCL11A expression in the erythroid lineage [11]. In the first clinical trial [NCT03432364; ST-400-01] by Sangamo Therapeutics and Sanofi, rapid hematologic reconstitution, HbF elevation and a persistence of editing rate was observed six months after transplantation in thalassemic patients, while SCD patients remained symptom-free for up to 52 weeks post CD34^+^-edited cell transplantation [12].

In immunodeficiencies, both inherited and acquired, ZFNs have been applied in several gene editing approaches. X-linked Severe Combined Immunodeficiency (X-SCID), characterized by mutations in the *IL2RG* gene and resulting in impaired humoral and cell immunity [13], was effectively corrected by the HDR-mediated editing of *IL2RG* via ZFNs, for both in patient-derived HSCs and xenotransplantation mouse models [14,15,16]. Wiskott-Aldrich syndrome (WAS), another rare X-linked congenital syndrome caused by mutations in the *WAS* gene and playing an important role in actin cytoskeleton remodeling specifically in HSCs [17], was also targeted by ZFN gene editing. ZFN-mediated introduction of the wild type WAS exons 2-12 in the first intron of the *WAS* gene resulted in a restoration of the protein expression (WASp) in patient-derived iPSCs, leading to an efficient differentiation of edited iPSCs into functional NK and T-cells [18]. In acquired immunodeficiencies, such as Human Immunodeficiency Virus (HIV) infection, the ZFN-mediated disruption of the *CCR5* locus—a principal receptor for HIV entrance into host cells—in CD4^+^ T-cells or in CD34^+^ cells significantly reduced the viral replication in non-human primates [19].

## 4. TALENS

Transcription activator-like effector (TALE) proteins are derived from the phytopathogenic bacterial genus *Xanthomonas* and are characterized by their DNA-binding ability. A wide range of effective domains such as nucleases, transcription activators or suppressors and site-specific recombinases can be fused with TALE proteins, supporting different genomic manipulations. Of these, the most common combination consists of the fusion of TALE-binding proteins with Fok1 nuclease to generate TALE nucleases (TALENs). Thus, TALENs comprise four functional domains: a nuclear localization signal (NLS), an acidic domain for target gene transcription activation, a central DNA-binding domain of 12-28 amino acid tandem repeats and a Fok1 nuclease [20]. Every single repeat within the DNA-binding domain consists of 30-35 amino acids and recognizes a single nucleotide respectively [21,22]. Given the fact that Fok1 nuclease has to be dimerized in order to generate a cleavage on both of the DNA strands, TALEN modules must be designed in pairs to bind opposite DNA target sequences [23].

TALENs have been used in β-hemoglobinopathies for the reactivation of γ-globin genes in a similar fashion to ZFNs. Specifically, 13nt deletion in HBG1 and HBG2 promoters via TALENs mimics a well-known, naturally occurring HPFH mutation resulting in HbF reactivation [24]. In addition, TALENs have been shown to inactivate the expression of BCL11A in CD34+ cells of non-human primates [25] or to correct the *HBB IVS-110(G>A)* mutation in human erythroblasts derived from *IVS-110(G>A)*-homozygous patients [26]. A targeted correction of the IVS2-654 C > T *HBB* gene mutation via homologous recombination using TALENs was demonstrated in β-thalassemia-derived iPSCs [27] and in a β-thalassemia mouse model [28]. Immunodeficiencies have also been targeted with the TALEN platform. Specifically, Menon et al. efficiently utilized TALENs in combination with a donor sequence to correct the *c.468+3A>C* mutation of the *IL2-Rγ* gene in patient-derived iPSCs [29], while Cellectis applied the TALEN^®^ technology to precisely correct RAG-1 mutations in preclinical studies [30]. In HIV, TALEN-mediated disruption of CCR5 in human CD4+ T-cells presented high specificity and efficacy against the infection [31,32].

## 5. CRISPR-Cas9

The discovery of the Clustered Regularly Interspaced Short Palindromic Repeats (CRISPR)-associated nuclease Cas9 (CRISPR-Cas9) system as a feature of adaptive immunity in bacteria and archeas led to a tremendous progress in genome editing. Τhe CRISPR-Cas9 system consists of a guide RNA (gRNA) complementary to a desired DNA sequence and a Cas9 nuclease [33,34]. After the recognition of the protospacer adjacent motif (PAM) at the 3′ end of the targeted DNA locus and the hybridization of gRNA with DNA sequence, the Cas9 nuclease is tied to the DNA-gRNA complex and generates a DSB on the specific locus which activates the endogenous DNA repair pathways [33,35].

The development of the CRISPR-Cas9 system has played a crucial role in the improvement of genome editing procedures for HSC gene therapy and the expansion of their potential application. In β-hemoglobinopathies, a generation of HPFH-associated mutations in the HBG promoters via a CRISPR-Cas9-mediated disruption of HbF suppressors-binding domains effectively increased HbF levels in both SCD and thalassemic patient-derived HPSCs, while it ameliorated the disease phenotype in xenografts [36,37,38]. Alternatively, the CRISPR-Cas9-induced disruption of HbF key regulators and their regulatory sequences (i.e., BCL11A, KLF1 and ZBTB7A), an approach already successful with ZFNs, was thoroughly investigated [39,40,41,42]. Several groups worked on the erythroid specific BCL11A disruption by targeting the aforementioned erythroid enhancer in the *BCL11A* locus, either individually or in combination with BCL11A-binding sites within the HBG1/HBG2 promoters [36,43,44,45]. In 2018, VERTEX Pharmaceuticals, in collaboration with CRISPR-Therapeutics, initiated two clinical trials for Transfusion-Dependent β-Thalassemia (TDT) [NCT03655678; CTX001-111] and Sickle-cell disease [NCT03745287; CTX001-121] based on ex vivo genome editing of BCL11a erythroid enhancer via CRISPR-Cas9 in CD34+ HSCs. Recently, disclosed results from the two studies at the EHA 2022 meeting demonstrated that 42 of 44 TDT-patients stopped RBC transfusions for up to 36 months, maintaining after month 3 mean total Hb levels >11 g/dL, of which more than 90% was HbF. SCD patients (n = 31) remained severe vaso-occlusive crises (VOCs)-free for a maximum duration of 32 months, having stable levels of approximately 40% HbF after month 4 [46]. Furthermore, Novartis has generated two gene editing products, OTQ923 and HIX763 [NCT04443907] for SCD, that reduce the activity of BCL11A thus resulting in HbF reactivation. Finally, two additional clinical trials, supported by Graphite Bio, Inc [NCT04819841; GPH101-001; CEDAR] and Editas Medicine, [Inc NCT04853576; EM-SCD-301-001] explored the CRISPR-Cas9 system for ex vivo editing of CD34^+^cells from SCD patients [47].

Fanconi Anemia (FA) has been a continuing challenge as a target disease for gene therapy over the years. A promising approach for gene therapy of Fanconi Anemia (FA) is the generation of compensatory mutations in the mutated FANCA gene with the CRISPR-Cas9 technology in order to restore its functions. Indeed, editing of the FANCA locus in HSPCs derived from FA patients through the introduction of compensatory mutations, led to the correction of cell phenotypes without affecting their differentiation and self-renewal capacity, as their transplantation into NSG recipients resulted in increased engraftment levels and a demonstrated in vivo proliferative advantage of edited cells [48].

The Wiskott-Aldrich immunodeficiency is caused by >300 different mutations of the *WAS* gene. Correction of each of these mutations with gene editing, although theoretically feasible, would have been impractical and expensive. Using the gene editing platform to knock-in a therapeutic WAS cDNA in frame with its endogenous translation starting codons in patient-derived HSPCs may ensure the correction of all known disease-causing mutations. Indeed, such HDR-mediated replacement of a mutated gene by a wild-type WAS cDNA via CRISPR-Cas9 led to the restoration of WAS protein levels and the improvement of T-cell functions [49].

As with the other gene editing platforms, the CRISPR-Cas9 system has also been extensively applied in HIV gene therapy. CXCR4, as well as CCR5, were efficiently disrupted in primary CD4^+^T-cells via CRISPR-Cas9 thus conferring resistance to HIV tropism [50,51]. Remarkably, the transplantation of allogeneic, CRISPR-edited, CCR5-ablated HPSCs into a patient with HIV-1 infection and acute lymphoblastic leukemia resulted in long-term engraftment of genetically modified HSPCs, yet with only 5% *CCR5* disruption rates in lymphocytes [52].

## 6. Double-Strand Break-Free Gene Editing

The evolution of CRISPR-Cas9 technology resulted in a tremendous optimization of genome editing approaches. However, concerns and limitations still exist; much attention has been paid to the unintended, “off-target” genotoxicity, which has now been well described while much less is known about “on-target” detrimental consequences arising from DSBs.

Off-targeting occurs due to nonspecific CRISPR/Cas-induced DNA cleavage at sites other than the actual target but with substantial sequence similarity to the intended target, thus potentially providing deleterious effects. In addition, DSB-induced activation of p53 with subsequent DNA damage response (DDR) and cell-cycle arrest has been described to decrease the HSPCs function and impair HDR, even though the delivery of Cas9 ribonucleoproteins has a limited lifetime within cells [53]. Transient p53 inhibition has been shown to enhance HDR efficiency, resulting in polyclonal reconstitution of the engrafted HSPCs [54,55].

In addition to the off-target effects, “on target” consequences include rearrangements and several kilo- to mega-bases telomeric deletions, ablation of entire chromosomes or the recently described chromothripsis, whereby massive genomic rearrangements occur in a one-off cellular crisis and can potentially generate a malignant outcome [56,57,58,59].

Collateral damage by gene editing may be permanent and irreversible. The accumulating evidence on the undesired either off-target or on-target consequences in clinically relevant primary cells warrants an optimization of the current editing systems and methods for genome-wide profiling of off-target effects and for decreasing or avoiding CRISPR off-target activity [60].

### 6.1. Base Editors

Gene editing strategies that do not generate DSBs, including base or prime editors (BE, PE), are considered substantially safer over traditional gene editing approaches as they overcome the DSB-associated deleterious effects in genome integrity [61]. Base editing precisely generates targeted point mutations without generating DSBs or requiring donor DNA templates and activation of the endogenous HDR mechanism [62].

*Cytosine base editors* (CBEs) consist of a cytidine deaminase fused to a mutated form of Cas9 from *Streptococcus pyrogenes* which is unable to generate DSBs (dead Cas9; dCas9). After binding to its target site, dCas9 performs local denaturation of the DNA complex to generate single strand DNA chains. Cytidine deaminase converts the desired cytosine to uracil in the strand which is not paired with guide RNA. Cell replication machinery recognizes uracil as a thymidine resulting in a C-G to A-T transition [63,64,65,66].

*Adenosine base editors* (ABEs). In contrast to DNA cytidine deaminases, there is not an enzyme catalyzing the deamination of adenine to inosine in nature. Up to date, all reported enzymatic inversions of adenine to inosine occur on free adenine, free adenosine, adenosine in RNA or adenosine in mis-paired RNA-DNA heteroduplexes [64,67]. To overcome this obstacle, Gaudelli et al. created an engineered enzyme based on a tRNA adenosine deaminase enzyme, TadA, originating from *Escherichia coli.* The recognition of adenosine by TadA results in hydrolytic deamination of adenosine. The remaining inosine is recognized by a cellular repair mechanism as a cytosine, and the intermediate T-I base pair is replaced by a G-C base pair [68].

Efforts to treat β-hemoglobinopathies with base editing have shown that targeting the +58 BCL11A erythroid enhancer with an A3A(N57Q)-BE3 efficiently converted the desired G-C to an A-T which led to the suppression of BCL11A expression and increased the levels of HbF [69]. Wang et al. used a hAPOBEC3A-Cas9n (hA3A-BE3) to introduce single nucleotide substitution at -115C and -114C in HBG promoter, mimicking HPFH mutations and increasing γ-globin expression from ~6.8% to ~44.2% [70]. Recently, an ABE- or CBE-mediated introduction of clustering mutations ~200 bp upstream of *HBG1/2* genes effectively reactivated γ-globin expression in SCD HSPCs via the disruption of LRF binding or the induction of KLF1 recruitment [71].

SCD is caused by a single base-pair point mutation (GAG to GTG) due to the replacement of glutamine acid (GAG) by valine (GTG), which could be an ideal base editor target. Nevertheless there is currently no base editor able to convert T to A. However, Newby et al. were able to generate an adenine base editor converting the GTG codon to GCG, leading to the expression of a non-pathogenic variant known as Hb-Makassar (HBB^G^) [72]. C. Li. et al. created an ABE in order to generate -113A>G mutation in the HBG promoter mimicking an HPFH mutation. In vivo editing of the promoter in β-YAC/CD46tg mice resulted in a 20% conversion rate in HSPCs and >40% γ-globin expression in peripheral RBCs [73]. The *HBB* -28A>G mutation is one of the most frequently detected mutations in β-thalassemia patients in China and East Asia preventing the transcription of the *HBB* gene [74]. Two base editor variants, the eA3A-BE and eA3A(N57Q)-BE3, were employed in erythroid precursor cells derived from a compound heterozygous thalassemia patient (4bp-deletion in exon 1 of one *HBB* allele and HBB -28 A>G mutation in the other), and they effectively generated the C>G substitution in HBB-28. Importantly, eA3A-BE or eA3A(N57Q)-BE3 editing increased HBB expression by 2.6 and 4.0-fold, respectively, compared to control samples [75].

The base editing technology has been applied in other disorders in addition to hemoglobinopathies. In particular, simultaneous disruption of the HIV receptors CXCR4 and CCR5, either in primary human T-cells via cytosine base editors or in primary T-cells, and CD34+ HSPCs via adenine editors efficiently disrupted CXCR4 and CCR5 expression thus protecting from CXCR4- and CCR5-tropic viral infections [76].

Adenine base editors have also been applied for the targeting of the FA-55 and FA-75 mutations in the *FANCA* gene resulting in the restoration of expression and phenotypic correction in HSPCs derived from a Fanconi anemia patient [77]. Finally, ABEs have been recently used for the targeted correction of CD3D C202T, a mutation causing CD3δ severe combined immunodeficiency (SCID) in Jurkat T-cells and in CD34+ HSPCs leading to a more efficient CD3 repair compared to a CRISPR-Cas9 correction via homologous recombination [78].

### 6.2. Prime Editing

Despite the enormous progress in precise genome editing with the discovery of base editors, the inability to install all possible combinations of bases substitutions represents a challenge that needs to be addressed. Recently, Prime Editors (PE), a versatile genome editing platform, has been generated [62]. The major advantage of prime editors is their ability to accomplish all twelve types of base pair conversions alone or in combination with the installation of small deletions/insertions in DNA, without the generation of DSBs. The prime editors (PEs) consist of a Cas9 nickase fused to an engineered reverse transcriptase (RT) [79,80]. The PEs use a prime editing guide RNA (pegRNA) which contains a sequence complementary to the desired DNA sequence, a prime editor binding site and the sequence that will be introduced to the genome after RT activation. Because PEs incorporate the edit in one of the two DNA strands, in order to manipulate the DNA repair mechanism to use the edited strand as a template for repairing the non-edited strand, an additional gRNA is used for creating a nick in the non-edited strand away from the initial nick [80,81].

### 6.3. Epigenome Editing

The epigenome is shaped by chemical compounds that modify or mark the genome being inheritable during cell division, albeit not part of the DNA itself. Thus, epigenome editing refers to the modification of the epigenome using engineered tools aiming to modulate the chemical state of DNA structure and function, representing an alternative way through which a cell’s phenotype and/or function can be altered without modifying the underlying DNA sequence [82].

In the last years, several studies have revealed the impact of epigenome alterations on gene expression and the development of genetic disorders and cancer as a result of the above. There are various epigenomic modifications that tightly control cellular processes [83]. DNA methylation is a major epigenetic modification known to cause a silencing of gene expression [84]. Another regulatory process is histone modification, which is induced by methylation and acetylation on histones located in the vicinity of promoters or enhancers. Histone methylation/demethylation are orchestrated by histone methyltransferases/demethylases, respectively, and histone acetylation/deacetylation are catalyzed by histone acetyltransferases (HAT)/deacetylases (HDAC), respectively [85,86].

The combination of the hitherto acquired knowledge in the field of epigenomic modifications with the innovative genome editing approaches has led to the generation of epigenome editors (epi-editors) [87]. The first epigenome editors were generated after fusion of the catalytic domains of enzymes such as DNMT3 to the catalytic residues of programmable DNA binding molecules (TALE, ZFP) [88,89]. Later, CRISPR epigenome editors were designed, consisting of a catalytically inactivated “dead” Cas9 (dCas9) fused or non-covalently bound to the catalytic domain of epigenetic effectors such as DNMT or TET enzymes, HATs or HDACs to activate or repress gene expression. A gRNA complementary to the target DNA sequence navigates the CRISPR-Cas9 epigenome editor to the target site, usually a promoter or distal cis-regulatory sequence [90,91]. Other epigenome editing tools transiently expressing transcriptional repressors, such as DNMT3A or a combination of the DNMT3a and KRAB domains to target the regulatory sequences of a gene of interest have been efficiently established. Specifically, inheritable targeted epigenome silencing was achieved in normal T-lymphocytes by inducing repressive histone marks and de novo DNA methylation [92]. Another system relying on the recruitment of Cas9 and transcriptional activation complexes to target loci by modified single guide RNAs has been developed to activate silenced endogenous target genes through trans-epigenetic editing. Proof-of-concept preclinical studies have shown that in vivo CRISPR/Cas9-mediated target gene activation (CRISPRa) improved phenotypes in relevant mouse disease models [93]. Moreno et al. recently coupled dCas9 with several transcriptional regulators, achieving multiplex targeting via single or dual-gRNA delivery that resulted in a high level of in vivo transcriptional repression (up to 80%) and transcriptional activation (up to 6-fold increase). This multiplex gene activation and/or repression approach could be beneficial for complex diseases that have multiple genomic loci involved [94]. CRISPRoff and CRISPRon are two technologies developed for programmable writing and erasing epigenetic memories; transient expression of CRISPRoff writes a robust, specific, and multiplexable gene-silencing program that is memorized by human stem cells through cell division and differentiation into neurons and can be rapidly reversed by CRISPRon [95].

Undoubtedly, epigenome editing-mediated transcriptional control may provide a platform for powerful and highly personalized therapeutics. Since this system does not rely on DNA breaks and genomic sequence modification, it is perceived to be reversible and less permanent, thus intrinsically safer. Nevertheless, the knowledge regarding the epigenetic editing effect in primary cells is limited, and basic biological questions still need to be addressed towards a safe translation to the clinic.

### 6.4. RNA Editing

The discovery of the Cas13 nucleases family, comprising 6 subtypes (a, b, c, d, X, Y) and having exclusively single-stranded RNA-targeting properties, further expands the range of genome editing approaches [96,97]. The unique feature of Cas13 nuclease is the RNase activity after an RNA-RNA recognition. The CRISPR-Cas13 system includes a CRISPR- RNA (crRNA) identifying a specific sequence on the target RNA. After the hybridization of crRNA with the targeted RNA sequence, Cas13 binds to the complex and cleaves in a specific position [98,99]. This approach leads to knock down of a specific gene without the interruption of a DNA sequence. Moreover, the substitution of Cas13 by catalytically inactive Cas13 (dCas13) can transform the CRIPR-Cas13 system into a programmable RNA binding tool.

The fusion of dCas13b with catalytic domains of RNA-modifying enzymes, such as the adenosine deaminase domain of adenosine deaminase acting on the RNA-2 (ADAR2) proteins, led to the conversion of adenosine to inosine, yet with a substantial number of off-target RNA-editing effects. The latter was addressed by further engineering of the system to create an ADAR2 variant capable of precise, efficient and highly-specific editing when fused to dCas13b [100,101]. Recently, Cas13 was harnessed to target known RNA viruses including lymphocytic choriomeningitis virus (LCMV), influenza A virus (IAV), vesicular stomatitis virus (VSV) and severe acute respiratory syndrome coronavirus 2 (SARS-CoV-2) [102,103]. Yin et al. showed that expression of CRISPR-Cas13a in HIV-effected cells successfully destroys viral RNA and prevents HIV reinfection in HEK293T human cell line, presenting an alternative potent approach for RNA editing in HIV infected T-cells [104].

## 7. Delivery Tools for In Vivo Gene Therapy

Most current HSC gene-addition and -editing clinical trials involve ex vivo manufacturing in which cells are harvested from a patient’s body by leukapheresis, engineered outside the body and then reintroduced into the patient, usually after a myeloablative conditioning. The ex vivo approach is highly sophisticated and cost-intensive while it requires transplantation expertise and infrastructure. In vivo gene therapy, namely the direct injection of the therapeutic vector into the patient in the absence of conditioning, has been extensively used for gene therapy of target tissues not easily accessible, such as liver, muscle, lung epithelium and neurons.

The bone marrow stroma generates a physical barrier to the transduction of HSCs with IV-injected gene transfer vectors, and this has been a major challenge for in vivo HSC gene therapy. Intraosseous (IO) delivery of hematopoietic cells has been explored as a means to mitigate the loss of cells given IV to high blood volume organs, such as the liver and the lungs. Although sustained transduction of hematopoietic stem/progenitor cells has been shown after lentiviral vector IO delivery post reduced intensity conditioning, the approach is technically challenging and invasive whereas it requires further optimization [105].

Based on the lessons gained during the evolution of gene-addition therapy especially as regards the in vivo gene delivery to non-hematopoietic tissues, spanning more than 3 decades of research and clinical translation, we will discuss the recent developments and challenges of in vivo gene therapy of HSCs.

### 7.1. Lentiviral Vectors

Lentiviruses, such as the human immunodeficiency virus (HIV), are RNA viruses of the *Retroviridae* family. HIV-derived lentiviral vectors (LVs) have been the gold standard for transgene delivery into HSCs due to their ability to transduce non-dividing cells and their safer genome integration pattern over γ-retroviral vectors [106,107] while they represent, up to now, the preferred tools for ex vivo gene correction. Self-inactivating lentiviral vectors with an enhanced safety profile have been used in numerous clinical trials for ex vivo HSC gene addition/editing, providing remarkable therapeutic benefits in patients with severe inherited blood disorders such as the Wiskott–Aldrich syndrome (WAS) [108,109], X-linked and ADA severe combined immunodeficiency (SCID-X1, ADA-SCID) [110,111], β-hemoglobinopathies [112] and neurodegenerative storage diseases (adrenoleukodystrophy and metachromatic leukodystrophy) [113,114].

For tissues with limited accessibility, such as the liver, in vivo lentivirus-mediated gene therapy specifically targeting the liver had been suggested from Luigi Naldini and Didier Trono early on, in 1996 [115]. However, LV-induced immune responses, by the in vivo administration, consisted of a major limitation leading to the reduction of transduction efficiency, the rejection of transduced cells and an overall inhibition of the therapeutic effect [116]. This effect was caused by the incorporation of the packaging-cell derived polymorphin class-I major histocompatibility complexes (MHC-I) in the virus’ surface. Packaging lentivirus in B2M-deficient cells can significantly reduce immune responses to the virus while not affecting viral titers and virus incorporation into the target cells [117].

LVs are modified to bear different viral envelopes (pseudotyping), most commonly the vesicular stomatitis virus glycoprotein (VSV-G), to alter and improve their tropism for different cell types. However, the inactivation of VSV-G pseudotyped particles by the human serum [118], as well as the lack of the LDL receptor in quiescent HSCs [119], represent obstacles for in vivo delivery of LVs to HSCs. Engineering the VSV-G glucoprotein to generate human serum-resistant and thermostable VSV-G variants [120], employing different envelops to pseudotype LVs (e.g., H/F-LVs, cocal LVs, measles and BaEV-LVs) [121,122,123] or using different retroviral vectors such as foamy viruses [124] represent approaches shown to achieve high-level transduction of unstimulated HSCs that was maintained in all hematopoietic lineages and in secondary-recipient NSG mice [125] or to successfully correct disease phenotypes by in vivo gene delivery (X-SCID) [121,122].

From a different perspective, the ability of LVs to accommodate relatively large transgenes, with a packaging size of about 10 kb, renders them ideal for the delivery of several genome editing tools in vitro or ex vivo. All the previously described editing systems can be comfortably fitted within the LVs with ample room for selection cassettes, donor templates or suicide genes. However, the stable, life-long expression of a DNA-editing protein delivered by the integrating to the genome LVs would pose a genotoxicity threat to the patients and possibly lead to intolerable toxicity. To overcome this issue, LVs depleted of their ability to integrate into the host genome (Integration deficient lentiviral vectors-IDLVs) or to reverse transcribe their transgene (non-reverse transcribable lentiviral vectors-NRTLVs) have emerged as a promising possibility [126,127,128].

### 7.2. Adeno-Associated Viral Vectors

Adeno-associated viruses (AAVs) are single-stranded, replication-deficient DNA viruses with a compact 4.7-kb genome flanked by palindromic inverted terminal repeats (ITRs). The AAVs represent a prominent tool for in vivo gene therapy for many diseases with >280 registered clinical trials world-wide [47] (ClinicalTrials.gov (accessed on 12 September 2022)), and several AAV-based therapeutic products (i.e., Glybera, Luxturna and Zolgensma) [129,130,131] have been approved by regulatory authorities. AAVs, like LVs, are capable of transducing both dividing and non-dividing cells [132], a requirement for in vivo HSC transduction. Their main advantage is that they are derived from viruses that have been evolutionary selected for transducing human cells, at the expense, however, of a human immune system response against these viruses and the vectors derived from these viruses, resulting in immune-mediated rejection after in vivo delivery [133].

Out of the wide AAV-serotype ranges available, AAV2 has been the first to be used in HSCs with positive outcomes [134,135,136], even though variable early results are attributed to differences in viral titers and donor variation [137,138,139]. In 2013, AAV6 was established as the optimal serotype to be used in human HSCs both in vitro and in vivo [140]. Subsequently, several groups have attempted to use AAV6 for the ex vivo delivery of a donor template for HDR-mediated targeting integration in HSCs [36,49,141,142,143,144], reaching an editing efficiency of 90% in vitro [145]. Even though delivery of a donor template through AAV6 has been largely effective, the use of AAVs as multiplex vehicles to deliver more complex and larger cassettes such as genome editing modules is challenging, mostly due to their low packaging ability.

ZFNs, with their monomer cDNA size approximately at 1kb, have been easily incorporated in AAVs since the early 2010s, either alone or in combination with a donor template for targeted integration [146,147]. However, encompassing larger sequences such as TALENs (3 kb/monomer), SpCas9 (4.2 kb), base (>5 kb) and prime (>6 kb) editors might prove challenging and require splitting the transgene cassettes in two separate vectors, a rather undesirable approach in an in vivo editing setting. As an alternative to AAV-spCas9 delivery, smaller Cas9 or Cas13 orthologs, such as sauCas9 (3.2 kb) or Cas13d [148,149] and CasRx (~3 and <4.3 kb, respectively), have been effectively incorporated in AAVs for DNA, RNA or epigenome editing [99,150,151,152].

Another major obstacle for the use of AAVs in vivo is the expected immune response and presence of neutralizing antibodies (NAb), as the wild-type AAVs infect humans at a young age without, however, developing any known pathology [153]. Studies in both small and large animal models described a prevalent serum neutralization of AAV6 transduction [154] while similar findings in humans for almost all serotypes have prompted the establishment of an NAb cut-off exclusion criterion for clinical trial enrollment [155,156,157,158]. Transduction inhibition due to neutralization, among other factors, created the need for higher viral doses which, in turn, were associated with acute liver toxicity. The latter, unfortunately, has led to the untimely death of four children and one young man in clinical trials of AAV8- and AAV-9-mediated correction of X-linked myotubular myopathy and Duchenne’s muscular dystrophy (XLMTM), respectively [159,160]. Acute liver failure was also the cause of death in two kids with spinal muscular dystrophy, 5-6 weeks after receiving the commercially approved AAV-9-based gene therapy product, Zolgensma [161].

Additional hurdles for AAV-mediated in vivo HSC gene therapy include the limited knowledge of AAV tropism to HSCs and their mainly episomatic cell localization, which, due to their unsuitability for multiplex manufacturing, rather precludes their use for targeting HSCs.

### 7.3. Adenoviral Vectors (Ads)

Adenovirus is a ~100 nm, non-enveloped, double-stranded DNA virus first isolated in 1953 [162]. Their ability to efficiently transduce a wide range of dividing and non-dividing cells, along with their well-known genome sequence, at early times led to the first in vivo gene delivery approach in an animal model [163]. The first human studies, however, demonstrated strong innate, humoral and cellular immune responses elicited by the Adenoviral vectors [164,165] which, in some cases, led to serious adverse events such as cytokine storm resulting in a patient’s death in 1999 [166]. Several strategies to circumvent these acute immune responses have been explored including the administration of immunosuppressive agents or monoclonal antibodies blocking cytotoxic T lymphocyte (CTL) immune responses [167,168,169,170,171,172].

To date, over 57 serotypes (Ad1-57) have been identified and divided in groups A-G. Of them, Group C Ad5 is the most well characterized and broadly tropic, albeit not towards HSCs. In contrast, Group B Adenoviruses, including Ad3, Ad11, Ad35 and Ad50 can transduce human HSCs via either desmoglein 2 (DSG2) [173] or the surface CD46 protein [174]. To harness the properties of Ad5 and the enhanced HSC transduction capabilities of group-B Ads, fiber-chimeric vectors containing B fibers on an Ad5 capsid were developed, and vectors incorporating the fiber of the CD46-tropic Ad35 (Ad5/35) were shown to efficiently transduce human HSCs [175,176] without causing liver toxicity after IV injection, as opposed to Ad5 vectors [177].

In addition to immunomodulating strategies to minimize the AdV vector-elicited immune responses, the development of 3rd generation “gutless” or Helper-Dependent (HDAd) vectors, deprived of all viral sequences, increased both the safety profile of the vectors and the efficiency and duration of the therapeutic effect in vivo [178,179]. In contrast to AAV, the large transgene capacity (>30 kb) of HDAds and their low manufacturing cost makes them ideal for the accommodation of any genome-editing modality or possible combinations.

Ads do not integrate their genetic material into the host cells’ genome. This feature would have precluded these vectors for HSC gene therapy which requires lifelong gene correction; however, recently, hybrid Hd5/35 vectors exploiting transposon-based transgene integration systems (*Sleeping Beauty*, *piggyBac,* and *Tol2*) [180] have successfully integrated transgenes into the genome [181,182].

A novel, simplified and minimally invasive platform for in vivo HSPC gene therapy using a hybrid vector system comprising a HDAd5/35++ vector with increased CD46 affinity for transgene delivery to primitive HSCs and a hyperactive Sleeping Beauty transposase (SB100X) for transgene integration has been developed by the lab of A. Lieber [183]. This platform consists of a mobilization round with G-CSF + Plerixafor followed by injection of the vector when the circulating HSPCs are in their highest concentration. Transduced cells home back to the bone marrow where they persist and stably express the transgene [182]. With this approach, which could be also applicable using other delivery methods, choosing the optimal mobilization scheme relative to the patient’s disease background comprises an important factor. For example, the G-CSF + Plerixafor combination is now considered to be superior to the standard G-CSF mobilization β-in thalassemia [184,185,186,187] and Fanconi Anemia [188], while G-CSF is contraindicated in patients with sickle cell disease and for SCD gene therapy, Plerixafor-mobilized HSCs are harvested [189,190].

The HDAd5/35++ in vivo HSC targeting platform has broad implications for gene addition and gene-editing therapy of inherited or acquired diseases that require high levels of therapeutic proteins in the blood circulation. Indeed, up to date, this approach has been shown to safely and efficiently transduce primitive HSCs or/and ameliorate or correct disease phenotypes either using stably expressing gene-addition systems for β-thalassemia, SCD, hemophilia [191], X-SCID [192] and SARS-Cov2 [182,191,193,194,195,196,197] or precision editing mainly for β-hemoglobinopathies and HIV [44,73,193,198,199,200] in human HSCs, mouse models and non-human primates [201]. This extensive work demonstrated that HDAds can comfortably carry several genome-editing systems in HSCs, such as ZFN dimers [198], CRISPR/Cas9 with one or more gRNAs [44] and base editors [73].

### 7.4. Non-Viral Transfer

Non-viral transfer offers an alternative delivery tool to overcome limitations associated with the viral vectors including the reduced packaging capacity (AAV) [202], the pre-existing humoral and cell immunity against certain viral serotypes leading to virus neutralization and reduction of in vivo transduction efficiency [203] and the increased risk of off-target effects and insertional mutagenesis with the prolonged presence of genome editing tools into non-dividing cells [204,205].

The non-viral gene-delivery methods are divided into two different categories: the physical methods such as electroporation, microinjection, hydrodynamic delivery and the chemical methods [206]. Herein, we focus on chemical methods which can be translatable in vivo. This category includes organic nanoparticles composed of lipid or peptide-based materials and natural or synthetic polymers. In some cases, inorganic agents such as calcium phosphate or metals are used for nanoparticle formation [207,208,209,210]. Until now, non-viral chitosan, Poly-(lactic-co-glycolic) acid (PLGA) and cationic lipid nanoparticles have been successfully applied in experimental protocols [211] as well as in gene therapy trials for cystic fibrosis [212,213]. Furthermore, organic nanoparticles have been used for targeting liver cells [214,215]. In a clinical trial for Transthyretin amyloidosis gene therapy [NCT04601051], lipid nanoparticles have efficiently been used for in vivo delivery of a CRISPR-Cas9 system resulting in transthyretin (TTR) protein disruption [216].

Although the non-viral delivery methods in HSC gene therapy are not widely applied, the replacement of viral platforms with chemical tools could overcome their associated side effects and decrease the cost of the procedure. PLGA nanoparticles, loaded with triplex-forming peptide nucleic acids (PNAs) and single-stranded donor DNA molecules introducing site-specific repair and recombination, were used to specifically modify either the *CCR5* gene or the *β- or γ-globin* gene in relevant mouse models in order to prevent HIV infection or correct the βIVS2-654 mutation, respectively [217,218]. Recently, Cruz et al. exploited PLGA-nanoparticles for CRISPR-Cas9 delivery into primary erythroblasts and human CD34^+^ HSCs to reactivate γ-globin expression [219]. The development of layer-by-layer (LbL) nanoparticles, which consist of a nucleic acid core, a negative charged layer and a non-degradable synthetic peptide, enhanced the delivery efficiency and made possible the targeted transport into HSCs. Specifically, LbL nanoparticles containing an outer layer of anti-CD117/c-kit antibodies attached to hyaluronic acid efficiently targeted in vivo mobilized HSCs in a mouse model [220].

## 8. Current Issues and Considerations for In Vivo Gene Therapy

The in vivo gene therapy has clearly several advantages as compared to the ex vivo approach. It is minimally invasive and simplified, thus abrogating the need for leukapheresis, myeloablative conditioning with chemotherapy and transplantation expertise as well as the barrier of limited patient accessibility to ex vivo gene therapy products due to their exorbitant costs. Moreover, by skipping the HSC ex vivo manipulation, the impaired homing/engraftment of transduced cells is avoided and all HSCs, including the “true” stem cells that could have been missed by isolating the HSCs on the basis of the “conventional” CD34^+^cell marker, can be targeted.

Undoubtedly, the future of in vivo HSC genome modification seems prosperous. However, there are still limitations that scientists should be able to overcome and explore before attempting this approach en masse. Vectors for in vivo HSC gene-addition therapy need to integrate into the genome and for in vivo gene editing to specifically and precisely bind to HSCs without off-target delivery. The primarily quiescent nature of HSCs makes in vivo gene targeting highly challenging, especially when nuclease-mediated HDR, restricted to the G2/S cell cycle phase, is considered and, in this context, NHEJ, base or prime editors should be more appropriate.

The optimal type of editor to be selected is crucial and reasonably high editing efficiency is a major prerequisite. However, efficiency must be coupled with retaining the modified cell’s fitness and function, minimal—if any—off-target effects and, of major importance, a low immunogenicity profile. In vivo gene therapy faces challenges from both the innate and adaptive immunity; pre-existing immunity and immunotoxicity represent a significant barrier for in vivo delivery, as several of the editing modules are of microbial nature and peptides of the programmable nucleases might be presented by Major Histocompatibility Complex (MHC) Class I molecules [221,222,223,224,225]. Pre-existing B- and T-cell responses against capsid proteins of Ad and AAV vectors can neutralize the vector before it transduces the HSCs and also prevent re-administration. Neutralizing antibodies against AAV1,2 and 6 and Ad5 [226] are prevalent in serum, whereas Ad35 is a rare human serotype and thereby Ad35 vectors evoke only mild host immune responses and contribute to prolonged gene expression [227]. Transient immunosuppression using corticosteroids with or without anti-IL-6R (tacrolimus) has been effective to confront humoral immunity in in vivo liver-targeted gene therapy for hemophilia B using AAV8 or AAV3 vectors [228,229]. Mobilization before IV vector administration may further increase cytokine release in response to an innate immune reaction. Nevertheless, pretreatment with dexamethazone, tacrolimus and anti-IL-1 to suppress the inflammatory cytokine storm allowed for safe in vivo gene editing in mobilized NHPs with the HdAd5/35 vector system [201].

Off-target effects might also differ significantly between nucleases and be also specific to the target locus. Detection of such off-target events is especially challenging with in vivo editing. It might be feasible though by an unbiased high-sensitivity deep whole genome sequence [230,231]. Recently, a dual step assay where the genomic loci harboring off-target events are identified in vitro and, subsequently, the same sequences are assayed in vivo has been described [232]. Additional methods for on- and off-target nuclease cleavage detection have been extensively reviewed by Andrew Atkins et al., 2021 [233]. In addition to the introduction of mutations in unpredicted or undesired genomic locations, double-strand brakes by programmable nucleases and especially CRISPR/Cas9 have been shown to cause large deletions and genomic rearrangements [234], chromothripsis [56] and aneuploidy [235].

Methods to mitigate current limitations are compulsory before broadly transferring these therapeutic approaches in the clinic. The continuously increasing developments in the field and the discovery of new, optimized and safer genome-editing modules and platforms may enable in vivo gene addition or editing and become clinically applicable, widespread accessible and an affordable treatment for all patients.

## Data Availability

Not applicable.

## Abbreviations

AAVs	Adeno-associated viruses
ABEs	Adenosine base editors
ADAR2	Adenosine deaminase domain of adenosine deaminase acting on the RNA-2
Ads	Adenoviral vectors
CRISPR-Cas9	Clustered Regularly Interspaced Short Palindromic Repeats CRISPR-Cas9 nuclease
CBEs	Cytosine base editors
CTL	Cytotoxic T lymphocyte
dCas9	Dead Cas9
DDR	DNA damage response
DSBs	Double-strand breaks
FA	Fanconi anemia
HbF	Fetal hemoglobin
HDAd	Helper-dependent adenovirus
HSC	Hematopoietic stem cell
HSPCs	Hematopoietic stem and progenitor cells
HPFH	Hereditary persistence of fetal hemoglobin
HDR	Homologous directed repair
HIV	Human immunodeficiency virus
IAV	Influenza A virus
ITRs	Inverted terminal repeats
LbL	Layer-by-layer
LVs	Lentiviral vectors lvs
LCMV	Lymphocytic choriomeningitis virus
MHC	Major histocompatibility complex
MMEJ	Microhomology-mediated end joining
Nab	Neutralizing antibodies
NHEJ	Non-homologous end joining
PNAs	Peptide nucleic acids
PLGA	Poly-lactic-co-glycolic acid
pegRNA	Prime editing guide RNA
PE	Prime editors
PAM	Protospacer adjacent motif
SARS-CoV-2	Severe acute respiratory syndrome coronavirus 2
ssODN	Single-stranded oligodeoxynucleotide
TALENs	Transcription activator-like effector nucleases
VOCs	Vaso-occlusive crises
VSV	Vesicular stomatitis virus
VSV-G	Vesicular stomatitis virus glycoprotein
WAS	Wiskott-Aldrich syndrome
XLMTM	X-linked myotubular myopathy and Duchenne’s muscular dystrophy
X-SCID	X-linked Severe Combined Immunodeficiency
ZFNs	Zinc-finger nucleases
